# Metabolic Regulation of Stem Cells in Aging

**DOI:** 10.1007/s40778-021-00186-6

**Published:** 2021-04-23

**Authors:** Andrea Keller, Tyus Temple, Behnam Sayanjali, Maria M. Mihaylova

**Affiliations:** 1 Department of Biological Chemistry and Pharmacology, College of Medicine, The Ohio State University, Columbus, OH, USA; 2 Comprehensive Cancer Center, Wexner Medical Center, Arthur G. James Cancer Hospital, The Ohio State University, Columbus, OH, USA

**Keywords:** stem cells, Aging, Metabolism

## Abstract

**Purpose of Review:**

From invertebrates to vertebrates, the ability to sense nutrient availability is critical for survival. Complex organisms have evolved numerous signaling pathways to sense nutrients and dietary fluctuations, which influence many cellular processes. Although both overabundance and extreme depletion of nutrients can lead to deleterious effects, dietary restriction without malnutrition can increase lifespan and promote overall health in many model organisms. In this review, we focus on age-dependent changes in stem cell metabolism and dietary interventions used to modulate stem cell function in aging.

**Recent Findings:**

Over the last half-century, seminal studies have illustrated that dietary restriction confers beneficial effects on longevity in many model organisms. Many researchers have now turned to dissecting the molecular mechanisms by which these diets affect aging at the cellular level. One subpopulation of cells of particular interest are adult stem cells, the most regenerative cells of the body. It is generally accepted that the regenerative capacity of stem cells declines with age, and while the metabolic requirements of each vary across tissues, the ability of dietary interventions to influence stem cell function is striking.

**Summary:**

In this review, we will focus primarily on how metabolism plays a role in adult stem cell homeostasis with respect to aging, with particular emphasis on intestinal stem cells while also touching on hematopoietic, skeletal muscle, and neural stem cells. We will also discuss key metabolic signaling pathways influenced by both dietary restriction and the aging process, and will examine their role in improving tissue homeostasis and lifespan. Understanding the mechanisms behind the metabolic needs of stem cells will help bridge the divide between a basic science interpretation of stem cell function and a whole-organism view of nutrition, thereby providing insight into potential dietary or therapeutic interventions.

## Introduction

One of the most accessible means of influencing lifespan has involved modulating caloric or dietary intake. Many early studies examining the effects of diet on age have focused on the abilities of caloric restriction, generally considered a 20–40% reduction in calorie intake on a whole, or restriction of specific dietary components such as protein or carbohydrates, to increase lifespan [1–4]. Diet can profoundly affect the onset of age-associated pathologies, such as cardiovascular disease, neurological impairment, immune dysfunction, metabolic syndrome, diabetes, and cancer [5, 6]. As human lifespan has increased globally over the last several decades [7], interest in improving healthspan, the time in life when an individual has limited chronic diseases, has also increased. Since then, regulation of diet by restriction of specific nutrients, such as carbohydrates, amino acids, or fats, as well as timing of nutrient intake, has garnered much interest due to such interventions modulating tissue growth, regeneration, and repair at the molecular level.

In many tissues, healthy state is maintained by populations of stem cells, whose metabolic requirements are varied and complex, and which often lose their self-renewal capacity with age [8–12]. Numerous studies in both vertebrates and invertebrates have shown that dietary restriction and manipulation can be either directly sensed by stem cells, or indirectly by the surrounding niche, and can subsequently dictate stem cell renewal, differentiation, and overall tissue homeostasis. Improving stem cell function through dietary interventions, or dietary mimetics, is an attractive target for extending both healthspan and possibly human lifespan. In this review, we will primarily focus on metabolic dependencies and regulation within intestinal stem cells, but we will also discuss several other populations of somatic stem cells, including hematopoietic, skeletal muscle, and neural stem cells, highlighting the effects of cellular and systemic age-dependent metabolic changes with respect to tissue regeneration. Finally, we will review how dietary interventions influence stem cell self-renewal and tissue regeneration.

### Age-Associated Changes in Somatic Stem Cells

Somatic stem cells are a unique population of undifferentiated cells that have the ability to self-renew and give rise to progenitors that subsequently replenish specialized cells within tissues. The rate at which adult somatic stem cells can proliferate and contribute to tissue regeneration and homeostasis can vary between tissues and with age. Perhaps the best studied and most defined adult stem cell population is hematopoietic stem cells (HSCs). HSCs are located in the complex niche microenvironment of the bone marrow [13] and have the ability to give rise to lineage-committed progenitors that replenish all specialized blood cell types throughout an organism’s life [14]. Seminal discoveries in the late 1950s and 1960s showed that the bone marrow contained a population of cells capable of repopulating the hematopoietic system and giving rise to myeloid and lymphoid lineages [15, 16]. Since then, sophisticated lineage tracing techniques, coupled with irradiation and transplantation experiments, have provided insight on long-lived cells within tissues that retain DNA labels and serve as reservoirs of adult somatic stem cells [17•]. In humans, with age, there is a shift towards increased myeloid cell production, and HSCs transplanted from aged mice into young, irradiated recipients show similar myeloid bias and reduced lymphopoieses [18].

In the 1970s, several influential discoveries were also made in epidermal stem cell biology, where long-lived epidermal cells could be maintained in culture over long periods of time and were shown to express specific keratin markers [19, 20]. Later, it was shown that epidermal cells can be expanded through culturing and engrafted successfully onto burn victims [21, 22]. Subsequently, label-retaining hair follicle stem cells (HFSCs) were also discovered and shown to contribute to hair follicle regeneration. With aging, HFSC numbers decline and exhibit age-dependent transcriptional changes in mice [23, 24••].

In the mammalian gastrointestinal tract, crypt base columnar cells were discovered in the 1970s and were later determined to be highly proliferative, Lgr5 high expressing (Lgr5^+^) intestinal stem cells (ISCs) that are present both in the small and large intestines. ISCs either self-renew or differentiate into progenitor cells, which themselves undergo several rounds of proliferation before differentiating into one of several intestinal cell types: secretory Paneth cells (the only differentiated cell type that travels downwards into the crypt), Goblet cells, enteroendocrine cells, Tuft cells, or the absorptive enterocytes which make up the majority of the intestinal lining [25]. In addition, the mammalian intestine also harbors a quiescent, “reserve” pool of stem cells at the +4 position in the crypt. These more slowly cycling stem cells have been shown to express a differential repertoire of stem cell markers like *Bmi*, *Tert*, and *Sox9* compared to the fast cycling, Lgr5^+^ cells [26, 27] and can also withstand radiation induced DNA damage better than high cycling Lgr5^+^ cells [28]. Even so, the number of functionally dividing ISCs per crypt is relatively low and reported to be around 5–7 cells each [29]. In *Drosophila,* which has been an excellent model organism to study the effects of diet on aging, intestinal stem cells express high levels of *Escargot* and give rise to enterocytes, enteroendocrine cells (EEs), and copper cells in the gastric region [30•]. The majority of *Drosophila* ISCs reside in the midgut and are primarily quiescent, unless the epithelium is challenged by injury or infection. With age, there is an increase of inflammation and gut dysbiosis, leading to hyperproliferation of ISCs, reduced tissue integrity, and barrier dysfunction [31]. Interestingly, gut intestinal health in *Drosophila* dictates overall organismal longevity, and compromised tissue homeostasis leads to barrier dysfunction and shortened lifespan [32].

It has been more than 20 years since Potten and colleagues showed that the aged mammalian intestine is less regenerative and has more apoptotic cells following irradiation [33, 34]. With the advent of transgenic lines that allow lineage tracing experiments, as well as ex vivo organoid cultures that can compare the function of stem cells from animals of different ages, there have been a plethora of recent studies characterizing morphological and functional age-associated changes in the mammalian gut. Nalapareddy et al. showed age-dependent diminished crypt numbers and slower proliferation rates in mice, which they largely attributed to diminished Wnt3a expression with aging [35••]. Supplementing organoid cultures of aged mice with exogenous Wnt3a overcame this age-associated deficit. Moorefield et al. also reported diminished organoid formation from aged animals, but an increase in cell numbers and proliferation of *Sox9-eGFP*-expressing reserve stem cells [36••]. With aging, Mihaylova and colleagues observed diminished proliferation in stem and progenitor cells, as well as diminished capacity for self-renewal in aged guts following irradiation [37••]. Similar to the previous two studies, they also reported lower frequency of formation of organoids from aged animals compared to 3- to 4-month-old mice [38••], attributing some of these changes to decreased fatty acid oxidation (FAO) in the crypts of aged animals. Dietary and pharmacological induction of FAO improved the function of aged ISCs. Pentinmikko and colleagues reported decline in stem cell function and showed that Notum, a secreted Wnt inhibitor that is specifically expressed in Paneth cells, is produced at higher levels in aged Paneth cells compared to young ones [39••]. They further showed that pharmacological inhibition of Notum in vivo improved clonogenicity of Lgr5^+^ stem cells and protected aged epithelium from DNA-damaging Fluorouracil (5FU) treatment. Igarashi and colleagues showed that supplementation of aged animals with NAD^+^ rejuvenates aged intestinal stem cells and restores stem cell numbers. In addition, aged crypts and stem cells had significant reduction of SIRT1 protein levels, and treatment with the NAD^+^ precursor nicotinamide riboside (NR) improved the recovery of DSS-treated aged mice [40]. Together, these studies suggest that there are age-dependent changes in ISCs numbers and function due to intrinsic and systemic metabolic changes, as well as key regulators of stemness such as Wnts. DNA methylation analysis on patient-derived organoids also recently revealed regional differences in epigenetic alterations with age, and importantly, these regional differences were maintained in organoid cultures. Epigenetic clock calculations using DNA methylation showed that aging of small intestinal crypts slows down with midlife [41••]. Interestingly, slower proliferation was observed and recently reported by Tomasetti et al. in human esophagus, duodenum, and colon, where they hypothesize it may be a possible protective mechanism to decelerate cancer incidence with age [42].

Multiple studies have also reported an increase in the number of secretory cells in aged intestines, particularly in the distal end of the intestine, suggesting potential skewing to secretory lineages [35••, 38••, 39••] with age. Gerbert and colleagues recently honed in on these potential region-specific effects in the intestinal tract by performing proteomic analysis on many sequential sections throughout the small intestine (SI) and reported a similar age-dependent increase of Paneth cell markers, reduction of Goblet cell numbers in the distal small intestine, and conversely, an increase in enterocyte markers [43••]. Collectively these studies suggest that there are age-dependent alterations in secretory lineage cells, likely in response to microbial or metabolite changes with age [36••, 43••]. We have further summarized these age-dependent phenotypes in Table 1, as well as interventions used in these studies to rejuvenate and restore the function of aged stem cells.

Other tissues also contain populations of somatic stem cells that show age-dependent functional changes, including the muscle and brain. With aging, there is a significant loss of muscle mass and strength, otherwise known as sarcopenia, which is a determinant of frailty. Although the list of proposed mechanisms for age-dependent muscle decline is extensive, and includes multiple hallmarks of aging, the role of muscle stem cells remains to be fully elucidated. In muscle, muscle stem cells (MuSCs) or satellite cells are the undifferentiated precursors to skeletal muscle cells and are located beneath the basement membrane of myofibers [44]. Fry and colleagues showed that depletion of satellite stem cells affects muscle regenerative capacity, without affecting sarcopenia [45]. In the mammalian brain, neural stem cells (NSCs), undifferentiated neural cells with the ability to differentiate into various glial and neuronal cells, are located within the subventricular zone where they can form neuroblasts that will travel to the olfactory bulb to form periglomerular and granule mature neurons [46]. As with other adult stem cell populations, NCSs lose their neurogenic ability with age, which may have numerous consequences. For a comprehensive review on age-dependent decline in neuronal stem cells, we direct our readers to a wonderful recent review by Negredo et al. [47•]. Some tissues also have the unique ability to respond to severe injury by evoking post mitotic cells to rapidly proliferate and give rise to more committed lineages to replenish injured tissue. This is the case of one regenerative response in liver due to injury. In addition, following severe toxin injury, the liver can also mobilize cholangiocytes which can give rise to mature hepatocytes [48].

## Metabolic Regulation of Adult Stem Cells in Aging and Dietary Interventions

### Molecular Mechanisms

Numerous studies in invertebrates and vertebrates have previously shown the beneficial effects of caloric restriction (CR) on increasing maximum lifespan in yeast, *C. elegans*, *D. melanogaster*, and mice [1, 2, 4, 49]. CR is thought to decrease the incidence of metabolic syndromes, cardiovascular disease, spontaneous cancers, autoimmune diseases, and neurological defects, while also improving insulin sensitivity in mice. The effects of CR on immune function are somewhat varied, but dietary restriction or cyclical fasting has generally been thought to rejuvenate the immune system, at least in the elderly [50, 51•]. Many of the beneficial effects of CR are seen through its influence on stem cell capacity for self-renewal, as discussed later, but early studies in humans have brought into question the feasibility of maintaining such a strict regimen of caloric restriction. Participants often report feelings of hunger and lethargy while on a CR diet, and they begin to exhibit decreased muscle mass and bone mineral density [6, 52]. As a result, many researchers have turned to other forms of dietary restriction, such as short-term or cyclical fasting, as well as restriction of specific components of the diet like amino acids, to study effects on lifespan.

Restricted protein diets have long been studied as a means of lifespan and healthspan extension, and these diets can have systemic effects on insulin signaling and glucose homeostasis. In *Drosophila*, feeding a low-protein, high-carbohydrate diet by specifically reducing the percentage of casein in dietary yeast leads to prolonged maximum survival [53]. Restriction of methionine in particular leads to an increase in lifespan similar to dietary restriction, but these effects may be more tied to the ratio of amino acids available [54]. However, aged mice (24 months) on a low protein diet begin to lose weight following 2 weeks on the diet [55], lending credence to the argument that protein restriction in the elderly may in fact be harmful. Mice on restricted protein diets have reduced adiposity, decreased blood glucose and insulin levels, and exhibit improved glucose tolerance even when fed an otherwise high-fat, high-sugar “Western” diet [56, 57]. Conversely, excess amino acids in the diet are associated with decreased lifespan and higher mutation rates in yeast [55] and *Drosophila* [53, 58]. Furthermore, in the study conducted by Solon-Biet et al., a high-protein, low-carbohydrate diet in mice was associated with glucose intolerance while a low-protein, high-carbohydrate diet actually exhibited decreased insulin levels and led to increased lifespan, although the authors do note that the types of carbohydrates consumed could have an influence on insulin sensitivity [58]. Restriction of the branched chain amino acids (BCAAs) leucine, isoleucine, and valine in particular leads to decreased blood glucose levels and improved insulin sensitivity, and extends the lifespan of male mice [59–61]. In mice and rats, methionine restriction alone can decrease blood insulin and glucose levels and potentially increase lifespan, and while the results may mimic those of caloric restriction, it is difficult to pin all of these positive effects on longevity on a single amino acid [62••–64].

**Fig. 1:**
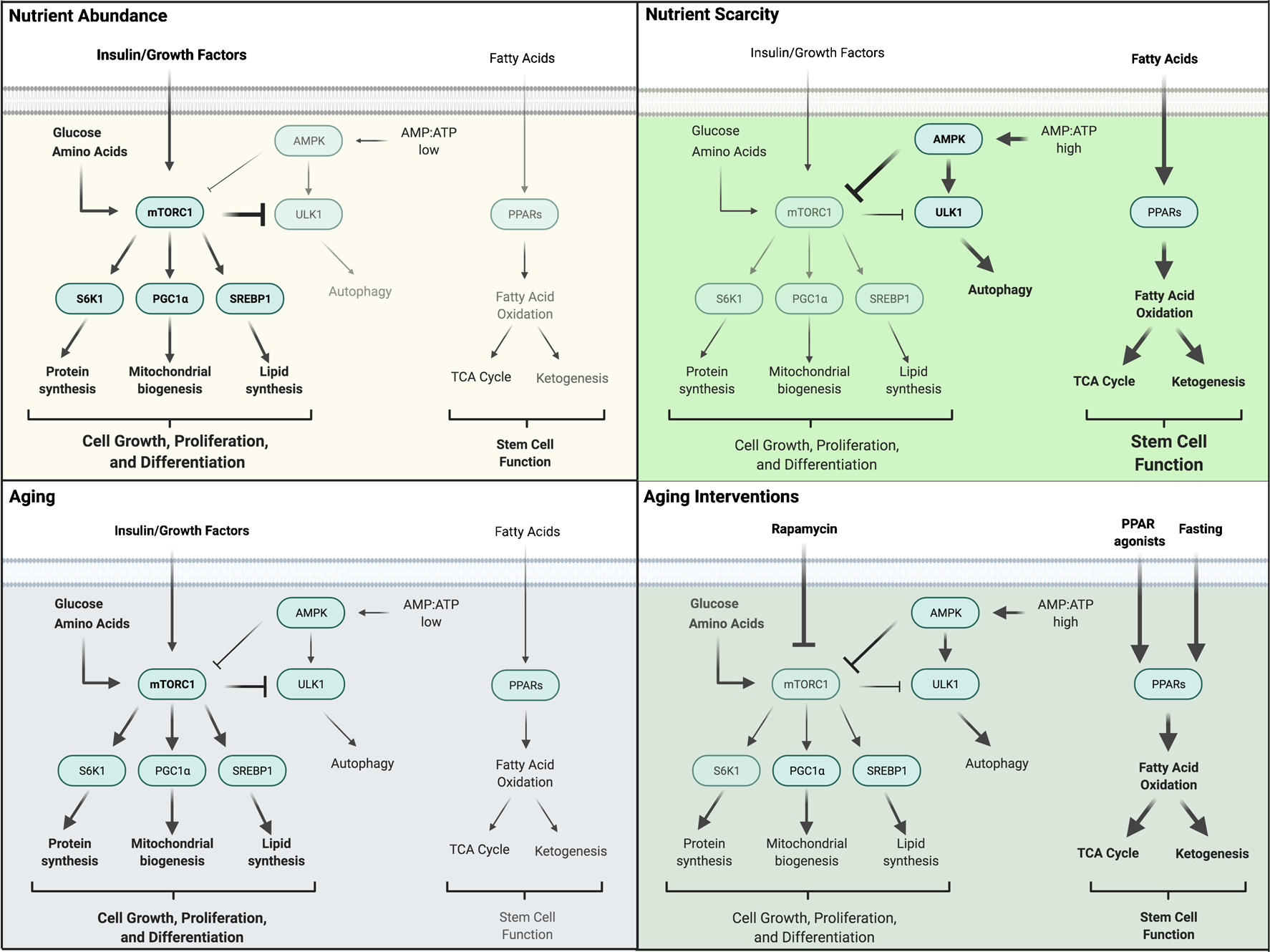
Nutrient sensing by the mTOR, AMPK, and PPAR signaling pathways. When growth factors and nutrients are abundant, mTORC1 is active and promotes anabolic processes, proliferation and differentiation, while AMPK and PPAR signaling are suppressed. When nutrients become scarce, AMPK is activated and mTORC1 becomes inhibited, resulting in increased catabolic processes and autophagy. At the same time, PPAR mediated fatty acid oxidation and ketogenesis are induced and enhance stem cell function, particularly in intestinal stem cells. With aging, fatty acid oxidation may be limited and mTORC1 activity may be hyperactivated leading to functional decline in stem cells. Interventions such as rapamycin aim to inhibit mTORC1, and treatments such as PPAR agonists or dietary regiments that enhance fatty acid oxidation and ketogenesis may improve age-associated decline in stem cell function, particularly in the gut

The mammalian target of rapamycin (mTOR) is often the focus of dietary studies like those described previously, as it specifically monitors amino acid availability, energy status, growth factor signaling, and oxygen levels (Fig. 1) [65–67]. Two separate complexes may form, mTORC1 and mTORC2, consisting of the serine-threonine kinase mTOR and a host of additional accessory proteins, which together serve as one of the primary means of sensing and responding to nutrient status in cells. When activated in times of nutrient abundance, mTORC1 promotes cell growth by phosphorylating ribosomal and translation initiation factors for protein synthesis, transcription factors associated with lipid synthesis, and coactivators involved with mitochondria biogenesis [65]. Additionally, formation of the autophagosome is inhibited through phosphorylation of the autophagy-initiating complex containing ULK1, Atg13, and FIP200 [68]. Inhibition of mTOR signaling using rapamycin extends the lifespan of yeast, *Drosophila*, *C. elegans*, and mice [69–72], although more recent studies are revealing that prolonged rapamycin treatment may have negative effects on certain tissues despite its positive effects on overall lifespan. It was previously believed that rapamycin only functioned to inhibit mTORC1 assembly, but work by Sarbassov and colleagues showed that prolonged rapamycin treatment also disrupts mTORC2 in multiple cell lines and tumors [73]. Lamming and others went on to show that rapamycin also disrupts mTORC2 in multiple tissues, leading to insulin insensitivity and glucose intolerance, and this mTORC2 disruption has negative effects on lifespan [74–79]. Genetic deletion of S6K1, a direct phosphorylation target of mTORC1, also leads to moderately increased lifespan, improved insulin sensitivity, and improved glucose tolerance [80], indicating that a balance must be found to maximize “beneficial” inhibition of mTORC1 and “harmful” disruption of mTORC2.

Like the mTOR complexes, the AMPK signaling pathway monitors nutrient status and regulates subsequent cell growth and differentiation processes by responding to changes in ATP, ADP, and AMP levels. In times of nutrient depletion, AMP and ADP concentrations rise, and AMPK is activated by phosphorylation of LKB1 at Thr172. This phosphorylation event is made possible through a conformational change induced by AMP/ADP binding at the regulatory γ subunit of AMPK. In turn, AMPK can directly and indirectly inhibit mTOR activity to reduce anabolic processes and promote autophagy [81]. Targets of AMPK include components of the mTOR complex (Raptor), transcription factors and enzymes that regulate lipid and fatty acid metabolism (SREPB1, ACC), and regulators of mitochondrial homeostasis (MFF) [82] among many others [83, 84]. High glucose levels can lead to decreased lifespan in *C. elegans* via inhibition of AMPK activity [62], whereas increased AMPK activity is correlated with increased lifespan, dependent upon correct balance of mitochondrial fusion and fission dynamics [85]. The beneficial effects of dietary restriction on longevity in *C. elegans* are in part dependent upon AMPK activity [86, 87]. Conversely, in instances of BCAA restriction, AMPK activity is upregulated through its phosphorylation, in contrast with the decrease in mTOR activity [59]. Several studies of dietary interventions, including metformin treatment or α-ketoglutarate supplementation, have found indirect effects on AMPK function. Such treatments ultimately alter the AMP:ATP ratio of the cell, leading to the activation of AMPK and the inhibition of mTOR [86, 88].

### Hematopoietic Stem Cells

Within the stem cell, changes in metabolism are able to direct various cellular phenomena such as senescence, proliferation, stemness, differentiation, apoptosis, and resistance to cancer treatment. HSCs remain in a largely quiescent state, with estimations of less than 10% to be actively dividing. Aging in the hematopoietic system is characterized by loss of self-renewal capacity of HSCs, expansion of HSC numbers, myeloid lineage skewing, and clonal hematopoiesis [89]. On the molecular level, these age-associated perturbations are thought to be largely driven by accumulated mutations and oxidative stress. ROS levels and redox state are important determinants of adult stem cell homeostasis in a number of tissues, and elevated ROS production is necessary for both differentiation and homing of HSCs to the bone marrow niche. Aged HSCs have increased ROS production [90], and a long-standing hypothesis was that age-dependent oxidative damage leads to aberrant phenotypes in HSCs. However, studies using accelerated mouse models of aging have shown that aging phenotypes in HSCs that occur due to mitochondrial dysfunction differ from HSCs from conventionally aged mice [91]. Another feature of aging in the hematopoietic stem cell system is clonal hematopoiesis of indeterminate potential (CHIP), a phenomenon characterized by reduced number of HSC clones that contribute to a large portion of hematopoiesis [89]. Recent studies have shown that with aging, many of the remaining HSC clones harbor mutations for important epigenetic enzymes, such as TET2 and DNMT3A, which leads to an increased risk of age-associated hematopoietic malignancies [92–94]. Another recent study showed that Kat6b, a histone modifier that is normally enriched in long-term HSCs, is suppressed in aged HSCs, which contributes to skewed production of myeloid cells [95••].

Hematopoietic stem cells must also maintain a balance of mTOR signaling over time. The activity of this pathway is linked to progenitor proliferation, yet inhibition of its activity with rapamycin in aged HSCs leads to improved stem cell function and renewal [11, 96]. Though once again, recent work is highlighting the different roles that these nutrient sensing pathways play in varying cell populations within a system, and in the niches in which they reside. For example, genetic loss of mTOR activity in the bone marrow microenvironment that informs HSC development can lead to aging phenotypes of both hematopoietic stem cells and progenitors [97]. Autophagy has also been shown to play an important role in HSC homeostasis and recently was shown to be impaired with age, leading to accumulation of active mitochondria and more metabolically active HSCs [98••].

Stem and progenitor cell function has also been shown to be regulated by mitochondrial metabolism and fatty acid oxidation [99]. Dome^+^ progenitor cells in the lymph glands of *Drosophila* use FAO as their primary metabolic state, unlike pre-progenitors and intermediate progenitors [100]. Kratchmarov et al. found that while FAO inhibition did not affect total conventional dendritic cell (cDC) or plasmacytoid dendritic cell (pDC) development, it significantly increased the amount of cDC2 cells and decreased cDC1 cells, suggesting that while anabolism drives progenitor division, catabolic mechanisms may push cells into alternative differentiation fates [101]. Similarly, AMPK-a1 deficiency both in vitro and in vivo did not change the cDC/pDC ratio but did change cDC differentiation toward favoring cDC2, suggesting that AMPK-a1 signaling may play a role in dendritic cell progenitor fate [102]. Regarding HSC exhaustion, Ma et al. found that Hes1 deletion upregulated both PPAR targets and FAO-related genes [103]. Inhibition of PPAR improved hematopoietic repopulation of Hes1-deficient HSCs, as did genetic deletion of CPT1A, a rate-limiting enzyme in fatty acid import into the mitochondria, suggesting that Hes1 regulates HSC hematopoiesis through suppression of PPAR-related pathways that otherwise would cause hematopoietic stem cell exhaustion. Caloric restriction has prevented age-associated decline of HSCs in certain mouse genetic backgrounds [104], but it does not prevent age-related decline of hematopoietic stem cell functioning in other studies [105].

### Intestinal Stem Cells

Numerous studies in *Drosophila* have informed how nutrient sensing and aging in the gut can regulate intestinal stem cells, tissue homeostasis, and longevity [106••]. Amino acid restriction leads to inactivation of mTORC1 in *Drosophila* [107], and further studies show that methionine restriction in particular can lead to lifespan extension, the effects of which are dependent upon mTOR signaling [62]. Early studies in the mammalian intestine have shown that it can respond to prolonged dietary restriction by decreasing villus length, indicating that nutrient availability can have direct consequences on absorptive surface area. More recent studies have shown how dietary restriction and fluctuations in nutrients can be sensed by the mammalian intestinal epithelium and can influence stem cell self-renewal and differentiation, as well as epithelial regeneration [9, 38••, 40, 43••, 108].

We and others have hypothesized that the beneficial effects of caloric restriction may be due to prolonged periods of fasting. To test this hypothesis, we recently asked how short-term, 24 hour fasting affects ISC function and numbers, especially in the context of aging. Interestingly, this short-term fasting led to a significant boost of both young and aged stem cell function and protected the intestine from radiation-induced damage. Unbiased RNAseq analysis of ISCs revealed fasting induced transcriptional upregulation of PPAR targets and subsequent metabolic switch to fatty acid oxidation. Pharmacological or genetic ablation of the rate-limiting enzyme for mitochondrial fatty acid import, CPT1A, blunted the effects of fasting in cultured organoids and reduced stem cell numbers in vivo, but treatment with PPARδ agonist GW501516 increased stem cell numbers, boosted stem cell function, and increased FAO rates in intestinal crypts from aged animals. Collectively, this suggests that FAO promotes stem cell self-renewal and that its disruption compromises ISC function [38••]. Long-term exposure to high fat diet (HFD) also drives a PPARδ-dependent program in intestinal stem cells and boosts stem cell numbers and function, while promoting niche independence of stem cells and increasing beta catenin activity. Importantly, chronic HFD exposure can enhance tumor-initiation capacity in progenitor cells deleted for the tumor suppressor *Apc* [79].

Cheng and colleagues recently showed that intestinal stem cells are highly enriched for HMGCS2, the rate-limiting enzyme in ketone body production, and that deletion of HMGCS2 in the intestine decreases beta-hydroxybutyrate (BOHB) levels in crypts, reduces stemness, and skews differentiation to a secretory lineage phenotype [109••]. Conversely, a high-fat ketogenic diet can enhance stem cell function in an HMGCS2-dependent fashion, where high levels of BOHB in ISCs act as endogenous HDAC inhibitors to regulate intestinal stem cell function and differentiation through Notch signaling [109••]. Gebert and colleagues also recently showed that dietary restriction followed by refeeding modulates HMGCS2 levels in the gut and influences secretory lineage differentiation [43••]. Meanwhile, ISC proliferation can also be regulated by cholesterol metabolism, where increased cholesterol biosynthesis can lead to increased organoid growth or ISC proliferation in vivo [110]. Another group has shown that a fasting-mimicking diet can modulate the microbiota and partially reverse inflammatory markers in dextran sulfate sodium (DSS)-induced colitis [111], and future studies in this area of research will shed more light on how diet influences the microbiome and its metabolic repertoire.

Even within the small intestine, there appears to be metabolic variability among stem cells in the intestinal crypt. Stine et al. found that both PRDM16 and PPAR expressions were highest in the duodenal crypts and decreased distally, suggesting duodenal stem cells rely more on fatty acid oxidation [112]. This was further supported by the finding that PRDM16 KO duodenal crypts contain lower numbers of proliferating cells and increased apoptotic cells in the transit-amplifying zone, suggesting that PRDM16 helps regulate epithelial cell renewal through FAO promotion [112]. Recently, it was also shown that loss of HNF4 leads to lower levels of FAO-related gene expression, and that knockout of HNF4a and HNF4g leads to increases in apoptosis, especially in the intestinal crypt base, indicating that HNF4 is necessary for stem cell survival. This was further supported through the supplementation of HNF4 DKO organoids with acetate, similar to levels present in the colonic lumen, which not only rescued the organoids from a spheroid morphology but also restored Lgr5-GFP-positive stem cells, showing that acetyl-CoA deficiency from the HNF4-related FAO defects could be limiting the renewal of stem cells in the intestinal crypt [113]. Interestingly, pharmacological inhibition of pyruvate metabolism or loss of mitochondrial pyruvate carrier (MPC) expression in Lgr5^+^ stem cells leads to increased stem cell proliferation, and a similar hyperproliferative phenotype was observed with the loss of *Drosophila* MPC [114]. Along the same lines, inhibition of MPC1 in hair follicle stem cells can induce activation and proliferation by promoting metabolism of pyruvate to lactate by lactate dehydrogenase [115].

With regards to mTOR signaling, in the aging mouse intestinal crypt, Pentinmikko et al. found that old Paneth cells exhibit increased mTORC1 activity, as evidenced by increased phosphorylation of the S6 ribosomal protein [39••]. Conversely, old intestinal stem cells show decreased pS6 levels compared to their younger counterparts, but do not maintain their capacity for self-renewal with age [38••, 40]. Indeed, current models point towards inverse trends in mTORC1 activation in Paneth cells and ISCs under the same treatment conditions [39••, 116], due to the fact that nutrient availability can be sensed by the niche and communicated to ISCs through paracrine secreted signals [9]. Caloric restriction increases stem cell numbers by suppressing mTORC1 signaling in niche Paneth cells, leading to increased levels of Bst1, an enzyme that converts NAD^+^ to cyclic ADP ribose (cADPR) to induce stem cell renewal [9]. Simultaneously, mTORC1 inhibition in ISCs due to CR promotes stem cell renewal and resistance to radiation-induced damage [117]. The interdependent relationship between ISCs and Paneth cells is further illustrated by the work of Rodríguez-Colman and colleagues, who showed that ISCs depend heavily on lactate produced by nearby Paneth cells to fuel their own mitochondrial oxidative phosphorylation, which leads to differentiation of ISCs [118]. As a result, the role of Paneth cells in regulating ISC function has garnered much interest from a metabolic perspective in recent years, and it is becoming more apparent that treatments such as rapamycin can have varied effects on different cell types even within the same niche, and thus varied ability to affect cellular aging.

Current studies are examining combination treatments that target pathways in addition to mTOR such as the Sirtuins, in order to account for these mixed responses across cell types and tissues. It is worth noting, too, that while rapamycin treatment alone can induce boosts in stem cell function and self-renewal that are generally seen with CR [9], treatment of crypts from calorie-restricted mice with rapamycin blocks these positive effects [40]. Thus, the influence of caloric restriction on intestinal aging is mediated in part by mTOR signaling, but is more complicated than simple mTOR inactivation.

In a similar fashion, AMPK phosphorylation is elevated in the intestinal crypts of calorie-restricted mice, and in the intestinal stem cells in particular [116]. As with mTOR signaling, the CR-enhanced self-renewal capacity of ISCs is blocked by inhibition of AMPK signaling in the ISCs, indicating that the beneficial effects of caloric restriction in ISCs are mediated through the activity of AMPK [116]. In intestinal stem cells as in hematopoietic stem cells, AMPK and mTOR together coordinate stem cell renewal, senescence, and even disease initiation with age, but the complexities of the relationship between fluctuating activities of each pathway are still being determined [119].

### Other Somatic Stem Cells

Like intestinal stem cells, neural stem cells exhibit marked decline in function with age and become less able to counteract neuron loss or damage with regeneration over time [47•]. Proliferative neural stem cells seem to turn to fatty acid oxidation when faced with mitochondrial dysfunction, shown through an increase in carnitine metabolites and the inability of palmitate to sustain cell viability without carnitine [120], suggesting that cells with mitochondrial dysfunction become more dependent on FAO to sustain cell proliferation. Yang et al. discovered that physiological levels of short-chain fatty acids, such as acetate, butyrate, and propionate, increased the growth rate of human neural progenitor cells that were derived from a human embryonic stem cell line, as well as encouraged the cells to undergo mitosis with no effect on apoptosis [121]. This suggests that short-chain fatty acids could regulate development of the neural system in early stages [121]. While treatment of mice with the gut hormone acyl-ghrelin did not increase proliferation in the subventricular zone, nor does it affect the number of adult-born neurons or the rate of neuronal differentiation, the increase of endogenous acyl-ghrelin through calorie restriction does increase the activation of new adult-born cells [122].

Knobloch et al. found that compared to proliferating neural stem and progenitor cells (NSPCs), quiescent NSPCs have higher expression levels of CPT1A. Inducing quiescence upregulates Spot14, a negative regulator of malonyl-CoA, suggesting that high levels of Spot14 along with low levels of malonyl-CoA help promote fatty acid oxidation. A massive die-off of quiescent NSPCs occurred after etomoxir treatment, while treatment of proliferative NSPCs led to a reduction of proliferation, indicating that FAO is much more crucial to quiescent NSPCs than proliferative ones, further supported through the upregulated expression of PPARα and its targets in quiescent NSPCs [123]. As an organism ages, neural stem cells also show a decline in mTOR signaling, which is believed to play a role in differentiation of stem and progenitor populations in the brain [96]. It has been found that induction of mTOR signaling promotes proliferation of NSCs in old mice, and in fact, that rapamycin treatment of young mice can generate NSCs with a more aged profile [124]. This provides another example that while rapamycin treatment appears to extend organismal lifespan on the whole, its tissue and cell specific effects must be considered in detail before it is more universally applied as an aging intervention. On the other hand, AMPK signaling is also implicated in the decline of neurological function with age, and its activity is elevated in old NSCs compared to young [125], yet some AMPK activity may be necessary for maintenance of autophagy and prevention of neurological disease [126].

In muscle, mTOR signaling is associated with muscle size, where its increased activity can contribute to hypertrophy but can also lead to muscle degeneration as a result of oxidative damage [127]. Consequently, age-associated muscle loss may be correlated with hyperactive mTOR activity in old muscle, and in rats sarcopenia can be prevented by treatment with rapamycin [127–130]. In contrast, the ability of skeletal muscle to activate AMPK appears to decrease with age in rats, and may lead to several age-associated impairments in mitochondrial function and lipid metabolism [83]. When treating satellite cells both in vivo and ex vivo with metformin, an AMPK agonist, Pax7 downregulation and the terminal differentiation of satellite cells are delayed, which is consistent with the inhibition of mTOR and reduction in RPS6 phosphorylation that induce a quiescent metabolic state [131]. Importantly, muscle satellite cells that maintain a more quiescent phenotype following metformin treatment have the potential to counteract age-associated muscle decline [131]. For both injured and non-injured control mice, there were significantly more satellite cells 1 week after injury in calorie-restricted mice than the control mice at 6 months of age; however, at 12 months of age, there were significantly fewer satellite cells in the calorie-restricted male mice than the control mice [132]. Treatment with metformin or administration of a calorie-restricted diet may induce AMPK activity in muscle and help to maintain the function of muscle satellite cells while preventing age-associated senescence [131, 133].

Satellite cells isolated from irradiated mice are more likely to have incomplete oxidation of 14C-palmitate and reduced levels of insulin-stimulated glucose uptake, regardless of whether mice were on a regular chow or high fat diet [134]. After observing impairments in the lipid profiles and increases in insulin resistance in childhood cancer survivors who had radiotherapy and chemotherapy, this, along with the experiments done in mice, suggests a metabolic reprogramming of satellite cells due to irradiation that could cause long-term metabolic dysfunction [134]. When using fetal and perinatal satellite cells along with post-injury myogenic cells, mitochondrial FAO genes had little change in gene expression while peroxisomal FAO genes were upregulated in activated cells. Satellite cells injured with notexin had significantly higher gene expression related to peroxisomal FAO than quiescent cells, which was not seen with mitochondrial FAO genes, suggesting different uses for the two FAO pathways in satellite cells. Culturing satellite cells with the peroxisomal FAO inhibitor, thioridazine, caused increases in levels of myogenin, while etomoxir inhibition of mitochondrial FAO did not, suggesting that inhibition of peroxisomal FAO induces differentiation in myogenic cells [135]. After culturing bovine satellite cells for 24 h with linoleic or oleic acid, expression levels significantly increased for genes such as *PPAR*α, *PPAR*γ, *ACOX*, *LPL*, *FABP4*, and *CPT1*, suggesting that unsaturated fatty acids like linoleic or oleic acids could promote FAO in muscle satellite cells [136].

## Conclusion

Somatic stem cells integrate critical environmental inputs that inform decisions on self-renewal, differentiation, and subsequent tissue turnover. Aging is a risk factor for many diseases, and recent studies are starting to uncover the molecular mechanisms of how environmental factors, such as diet, can influence stem cell behavior over time. With aging, many adult stem cell populations accumulate damage and become impaired in their function, which leads to inefficient tissue repair and may predispose to age-associated diseases such as cancer. Although numerous studies have shown that dietary restriction confers beneficial effects on overall organismal lifespan, we are just starting to uncover the complexities of metabolic dependencies in stem cells and how the availability of specific nutrients are sensed within a heterogeneous population of stem, progenitor, and niche cells and communicated between each other. The advent of new single cell technologies has already begun to enhance our ability to resolve the complex metabolic heterogeneity and interactions that exist in certain niches, such as in the gut crypt and bone marrow.

Recent studies using single cells technologies have also revealed that seemingly uniform, terminally differentiated cells in the liver and intestinal villus have specific transcriptional metabolic profiles that are driving cell function. These differences among hepatocytes and enterocytes were largely influenced by location, proximity to nutrients, and oxygen supply within their respective tissues [137, 138]. In the upcoming years, as multiple single cell-omic technologies advance and integrate, we will begin to see more advanced tissue maps of transcriptional, proteomic, and metabolomic signatures of stem and progenitor cells and their corresponding niches, both under homeostatic conditions as well as during aging and other pathological states. These types of studies will also likely layer dietary patterns with other environmental factors to expand our understanding of how nutrients and systemic metabolism impact tissue homeostasis. Finally, the constant improvement and engineering of primary 3D organoid cultures will allow us to more precisely examine intrinsic and extrinsic age-associated changes, as well as measure the activity of metabolic pathways, in response to defined nutrient conditions. We will be able to incorporate and study other signals in these systems, such as cytokines, hormones, and microbial metabolites. All of these advances will conceivably lead to better strategies and therapies for tissue repair with age, while carefully avoiding interventions that may accelerate age-dependent diseases such as cancer.

## Figures and Tables

**Table 1:** Age-associated changes in the intestinal crypt

Study	Model organism	Observed age-associated changes	Intervention

Martin K et al., 1998a [34], Martin K et al., 1998b [33]	*M. musculus*	Crypt area increase in distal SI Number of crypts decreased with aging in proximal and distal SI	None
		Less regeneration with irradiation in aged animals	
		More apoptotic cells in aged intestine	
Kozar et al., 2013 [29]	*M. musculus*	Continuous clonal labeling	None
		Number of stem cells and rate of replacement do not change up to 2 years	
Nalapareddy et al., 2017 [35••]	*M. musculus, H. sapiens*	No changes on stem cell numbers Increase in crypt length and width	Wnt3a supplementation in culture
		Increased number of Paneth Cells	
		Reduction of Wnt3a expression	
		Increase in villi length	
		Decreased proliferation in crypts	
		Fewer organoids/crypt in culture	
		Lower Wnt3a mRNA	
Moorefield et al., 2017 [36••]	*M. musculus*	Examined Sox9-positive reserve cells	None
		Increase in villus length	
		Increase in Paneth cells	
		Increased apoptosis in crypt	
		Fewer organoids/crypt in culture with aging	
Mihaylova et al., 2018 [38••]	*M. musculus*	Decrease in stem cell numbers Increase in Paneth cell numbers in proximal and distal SI	Short-term fasting (24-h complete food withdrawal) PPAR agonist GW501516
		Less functional aged stem cells	Exogenous lipids in culture
		Fewer organoids/crypt in culture with aging	
		Reduction in Olfm4-positive cells	
		Reduced crypt proliferation	
		Lower number of surviving crypts post irradiation	
Pentinmikko et al., 2019 [39••]	*M. musculus, H. sapiens*	Increased number of Paneth cells Fewer organoids/crypt in culture with aging	Notum inhibitor Rapamycin
		Reduction in Olfm4-positive cells	
		No change in Edu^+^ cells with ageing	
Igarashi et al., 2019 [40]	*M. musculus*	Higher Paneth cells numbers	Nicotinamide riboside (NR)
		Increase in villus length	
		Less crypt proliferation	
		Reduction in Olfm4-positive cells	
		Fewer organoids/crypt in culture with aging	
